# Association Between Pandemic Fatigue and Disease Knowledge, Attitudes, Concerns, and Vaccination Intention at Two Key Moments of the COVID-19 Pandemic

**DOI:** 10.3389/ijph.2023.1606049

**Published:** 2023-09-26

**Authors:** Adriana Agurto-Ramírez, Candela Pino-Rosón, Alba Ayala, María Falcón, Carmen Rodríguez-Blázquez, Maria João Forjaz, María Romay-Barja

**Affiliations:** ^1^ Hospital General Universitario de Valencia, Valencia, Spain; ^2^ Centro Nacional de Medicina Tropical, Instituto de Salud Carlos III, Madrid, Spain; ^3^ Departamento de Medicina Preventiva e Saúde Pública, Universidade de Santiago de Compostela, Santiago de Compostela, Spain; ^4^ Unidad de Investigación en Cuidados y Servicios de Salud (Investén), Instituto de Salud Carlos III, Madrid, Spain; ^5^ Research Network on Chronic Diseases, Primary Care and Health Promotion (RICAPPS), Madrid, Spain; ^6^ Departamento de Ciencias Sociosanitarias, Medicina Legal y Forense, Instituto Murciano de Investigación Biosanitaria (IMIB), Universidad de Murcia, Murcia, Spain; ^7^ Centro Nacional de Epidemiología, Instituto de Salud Carlos III, Madrid, Spain; ^8^ Centro de Investigación Biomédica en Red sobre Enfermedades Neurodegenerativas (CIBERNED), Madrid, Spain; ^9^ Centro de Investigación Biomédica en Red de Enfermedades Infecciosas (CIBERINFEC), Madrid, Spain

**Keywords:** COVID-19, knowledge-attitude-behaviour, health behaviours, preventive practices, pandemic fatigue

## Abstract

**Objective:** This study aimed to describe the change in knowledge, attitudes, concerns, perceptions, preventive practices, and vaccination intention at two key time points of the COVID-19 pandemic and to assess whether these changes varied by level of pandemic fatigue.

**Methods:** Data included in this study came from the third and the ninth round of the COSMO-Spain cross-sectional study. A general linear model was used to investigate the interaction terms between rounds and levels of pandemic fatigue.

**Results:** Changes between rounds were observed in knowledge, attitudes, concerns, perceptions, behaviours, and vaccination intention. Significant interactions between rounds indicated that those with low levels of pandemic fatigue had a greater increase in knowledge, lower decrease in concerns, greater decrease in agreement with the decisions made, and lower increase in vaccination intention compared with those with high pandemic fatigue.

**Conclusion:** As a pandemic evolves, it becomes necessary to consider the level of pandemic fatigue of the population and how this affects knowledge, concerns, and agreement with the measures adopted, as they influence the population’s adherence to public health recommendations aimed at controlling infections and protecting the most vulnerable.

## Introduction

Coronavirus 2019 disease (COVID-19), declared a pandemic in March 2020 [1], has produced more than 664.7 million cases around the world [2]. The main preventive measures for control of the pandemic were based on non-pharmacological interventions (NPIs) that imply population behaviour changes, such as social distancing, hand hygiene, and the use of facemasks. Most restrictive measures have involved significant changes in the population’s daily life, including isolated periods, quarantines, and mobility and social iteration limitations [3].

Despite adherence to these measures having saved many lives, these restrictions have also negatively impacted our life in many aspects such as economic stability, employment, mental health, and access to medical assistance [4], resulting in a decrease in the population’s well-being and affecting mental health [5]. Throughout the pandemic, most countries have adapted preventive measures according to their epidemiological situation [5], but maintaining a high degree of adherence over time and keeping the people aware about the pandemic has been a demanding task, often resulting in a loss of strength to carry it out.

These feelings of tiredness about the pandemic are a matter of concern and the World Health Organisation defined “pandemic fatigue” as the demotivation to follow recommended protective behaviours and proposed a framework on how to “maintain and reinvigorate” people’s motivation to comply with policies in response to COVID-19 [6]. In order to be able to measure pandemic fatigue, Lilleholt et al. [7] developed the COVID-19 Pandemic Fatigue Scale (CPFS) with two dimensions: people’s intention to comply with recommended heath-protective behaviours and their intention to be informed about the pandemic. This scale was validated in Spanish, providing researchers and other professionals with a valid and reliable instrument to explore the potential predictors and consequences of the pandemic fatigue [8].

The factors most frequently found to be associated with pandemic fatigue are age, sex, education level, and employment status [9], along with other contextual factors such as the number of COVID-19 waves, the tightness of the restrictions, prevalence of COVID-19, and disease severity [10]. The knowledge, attitudes, and perceptions of the population about COVID-19 have been changing throughout the pandemic [11]. Maintaining them at the desired level over time is a demanding task, often resulting in a loss of strength to continue such maintenance. Conversely, in order to design effective public health interventions, it is important to understand whether pandemic fatigue has in turn affected the population’s knowledge, attitudes, concerns, perceptions, preventive practices, and vaccination intention. Assessing the influence of pandemic fatigue on these factors is an essential point in public health that may determine how to motivate the necessary resilience of the population to comply with policy response.

However, most studies have focused on the factors affecting pandemic fatigue and how this fatigue influences adherence to non-pharmaceutical interventions [12], and there is little evidence regarding how pandemic fatigue may affect other aspects such as population knowledge, attitudes, concerns, and intention to be vaccinated.

The aim of the study was to describe the changes over time in knowledge, attitudes, concerns, attitudes, perceptions, preventive practices, and vaccine intention towards COVID-19 in the Spanish population at two key moments of the pandemic (just before the start of the vaccination campaign and 1 year later), taking into account individual and contextual factors, and to assess then which of these changes observed were influenced by pandemic fatigue. This study may help to design public health strategies by strengthening those aspects that may be affected by the effects of pandemic fatigue in the population.

## Methods

### Sampling and Data Collection

**FIGURE 1:**
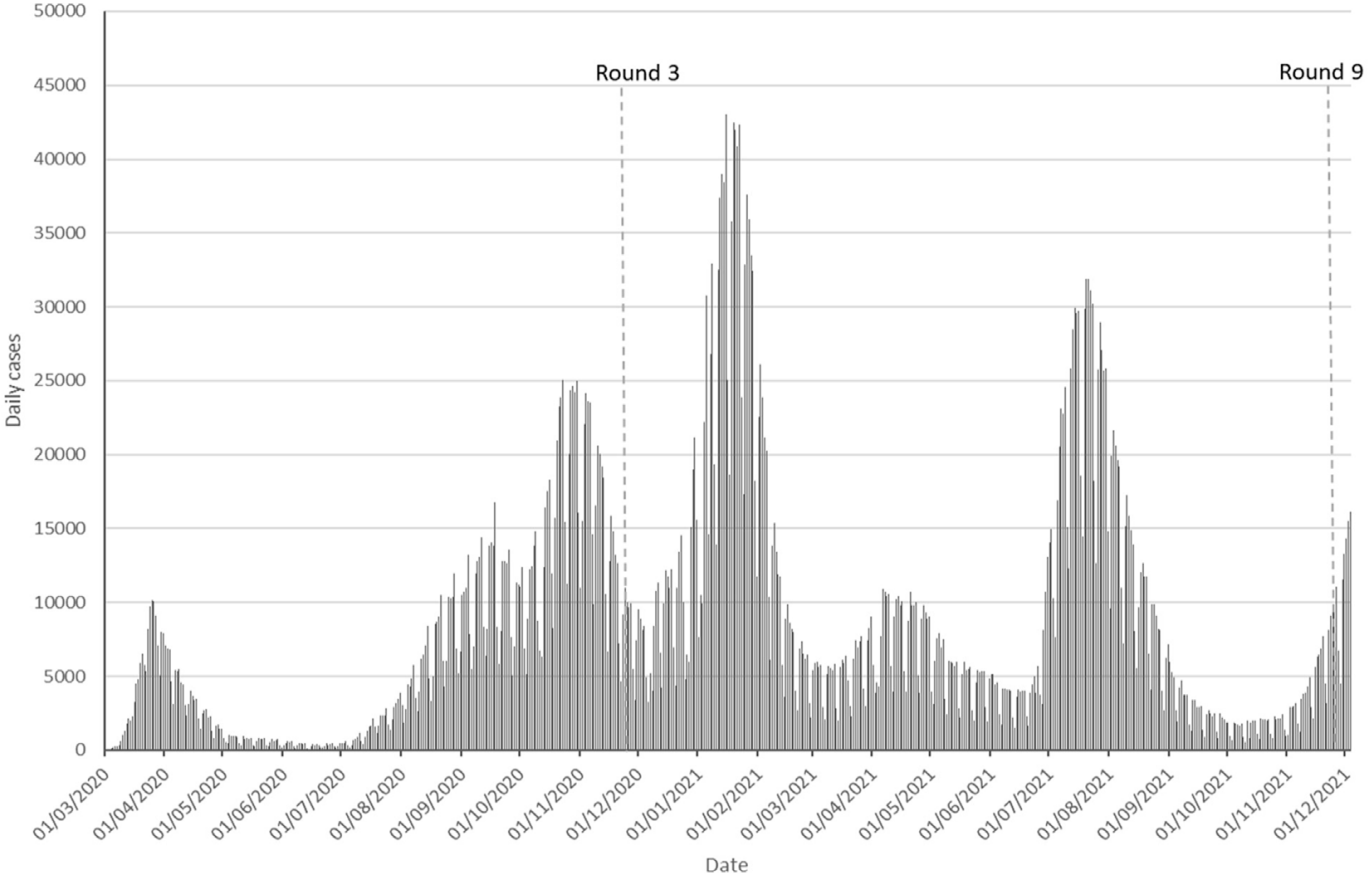
Incidence of COVID-19 daily cases in Spain from March 2020 to December 2021.

Data included in this study came from the third and the ninth round of the COSMO-Spain cross-sectional study, which has been conducted every 2 months since July 2020 [13]. The COSMO-Spain study survey is conducted by a consumer research company recruiting around 1,000 participants in each round. Participants aged 18 years and over are stratified by sex, age, geographic area, and educational level, representing the general Spanish population. The main objective of the COSMO-Spain study is to monitor the knowledge, attitudes, concerns, behaviours, and perceptions of the population during the COVID-19 pandemic. Round (R3) was conducted in November 2020 (*n* = 1,018) and the ninth round (R9) was conducted a year later, in November 2021 (*n* = 1,049) (Figure 1).

### Epidemiological Situation

In November 2020, Spain declared 1,628,208 confirmed COVID-19 cases, with a cumulative incidence of COVID-19 cases of 265.76 per 100,000 inhabitants for the last 14 days. COVID-19 hospital occupancy was at 11.52%, and critical care units were at 26.30% of their occupancy [14]. Vaccination was not yet available, and the estimated prevalence of SARS-CoV-2 IgG antibodies was 5.2% [15].

One year later, the cumulative incidence of COVID-19 cases for the previous 14 days was 323.07 per 100,000 inhabitants. Hospital occupancy was at 4.48% of COVID-19 cases and critical care units were at 11.41% of their occupancy [16]. More than 89.0% (37,615,143 people) of the target population had received the complete vaccination schedule of two doses, and 91% of the target population had received at least one vaccine dose [17]. Given the above-mentioned epidemiological changes and vaccination rates from one round to another, it seems appropriate to analyse the factors associated with these changes. Furthermore, many restrictive measures were in place during Round 3 in November 2020 while, 1 year later, most restrictive measures were already relaxed (Table 1).

**TABLE 1:** Main mandatory measures implemented by rounds (Spain, 2020–2021).

	Round 3 November 2020	Round 9 November 2021
Use of facemasks	Mandatory indoors and outdoors	Mandatory only indoors
Capacity of public spaces	Restricted	More flexible
Private gatherings	Restricted	Allowed
Curfews	Mandatory throughout the country	Mandatory by region
Internal travel restrictions	In force	Not in force
Social-health centres	Visits limitations	Visits allowed
COVID passports	Not yet available	Mandatory in most regions

Source: Ministry of Health BOE-A-2020-11590. Sec. Section III Oct 1, 2020, p. 83224; State strategy against the second wave. Ministry of Health; 2020 [38]. Nov Available at: https://www.sanidad.gob.es/profesionales/saludPublica/ccayes/alertasActual/nCov/documentos/Estrategia_estatal_segunda_ola.pdf; Law 2/2021, of March 29, on urgent prevention, containment and coordination measures to deal with the health crisis caused by COVID-19. Available at: https://www.boe.es/eli/es/l/2021/03/29/2/con (16–18).

### Variables

Socio-demographic data (sex, age, education level, job, and COVID-19 infection status) were collected in both rounds. COVID-19-related knowledge and attitudes (feeling depressed and feeling fearful, perception of the pandemic situation, and level of agreement with the decisions made), the population’s concerns about the disease (COVID-19 level of worry), risk perception (probability of becoming infected, disease severity, self-efficacy), preventive practices (adherence to preventive practices, frequency of consulting information about coronavirus) and intention to be vaccinated were included. Data collection followed the parameters indicated in the WHO COVID-19 Snapshot Monitoring survey protocol [18], facilitating comparability among more than 31 participating countries. The study protocol is available in the PsychArchives database (www.psycharchives.org/handle/20.500.12034/4313).

The COVID-19 Pandemic Fatigue Scale (CPFS) [7, 8]: (Cronbach-alpha = 0.74) was used as an independent variable including its six items: tiredness of COVID-19 discussions, feeling strained from following regulations, feeling sick of hearing about COVID-19, tired of restraining oneself, avoiding talking about COVID-19, and losing the spirit to fight. Responses ranged from 1 to 5 (1 for “strongly disagree” to 5 for “strongly agree”). A dichotomous variable (low and high fatigue) was created using the mean as the cut-off point. The scale has been translated into Spanish and validated for the Spanish general population [8].

Knowledge level index: An index from 0 to 7 points was obtained by adding the values of the seven correct statements rated with a value of 1 or 0, where 1 corresponds to “yes” and 0 to “no”. These statements were related with COVID-19 transmission, symptoms, appropriate application of preventive measures, and adequate behaviour in case of symptoms.

Concerns scale: A scale ranging from 10 to 50 points (Cronbach’s alpha = 0.82) was obtained by adding the values of the 10 component items. These items, rated with a score from 1 to 5, where 1 corresponded to “nothing” and 5 to “a lot,” included concerns about losing a loved one, hospital overcrowding, labour reconciliation, losing the job, having financial problems, not wearing a mask, going outside, a new lockdown, physical and mental health, and intra-family disputes to comply with the preventive measures.

Attitudes toward the decisions taken: This scale is made up of the sum of the scores (1 = “I do not agree at all” to 5 = “I totally agree”) of the different items (masks being mandatory or not outdoors, freedom of movement between countries and between provinces, the end of curfews, opening of educational establishments, etc.). In this scale, the items differ from round to round according to the restrictions and measures put in place to control the pandemic. A linear transformation of the scores was performed on a scale from 0 to 100 (Cronbach’s alpha = 0.74).

Adherence to preventive measures scale: A scale ranging from 11 to 55 points (Cronbach’s alpha = 0.84) was obtained by adding the values of 11 items rated with a score from 1 to 5, where 1 corresponds to “never” and 5 to “always”. The items assessed the frequency of the implementation of preventive measures in the last 7 days (hand washing with soap and water for at least 20 s, the use of hydroalcoholic gel for hand washing, not attending gatherings of friends or relatives, social and physical distancing, the use of masks as recommended, ventilation of closed spaces, disinfection of surfaces, avoiding public transport and crowded places, avoiding visits of relatives or friends during quarantine, and the use of masks at the homes of relative or friends).

### Data Analyses

Descriptive analysis was applied to all variables. Frequencies and percentages were used to summarise the data. The Chi-square test for categorical variables or Mann–Whitney U test for non-normal continuous variables were performed to assess the differences between rounds. A general linear model (GLM) and logistic regression models were developed, obtaining the estimated regression coefficients (*β*) and the corresponding statistical significance (*p*-value <0.05) for those dependent variables not included in the pandemic fatigue definition: COVID-19-related background, attitudes towards COVID-19-related policies and interventions, risk perception, population concerns, and vaccination intention. The independent variable was pandemic fatigue (dichotomous variable). Models were adjusted by sex, education level, job, and age, including the interaction term between round and level of pandemic fatigue (Eq. 1).

$$y=\beta_{0}+\beta_{1}∙x_{1}+\beta_{2}∙x_{2}+\beta_{3}∙x_{3}+\beta_{4}∙x_{4}+\beta_{5}∙x_{5}+\beta_{6}∙x_{6}+\beta_{7}∙x_{5}*x_{6}$$ (1)

where x_1_ = sex; x_2_ = education level; x_3_ = job; x_4_ = age; x_5_ = pandemic fatigue; x_6_ = round; and x_5_∗ x_6_ = interaction term between pandemic fantigue and round.

Margin plots were constructed for those models with significant interactions (*p*-value <0.05). The analysis was performed with the IBM SPSS software version 28.0.1.0.

### Ethic Statement

This study was approved by the Ethics Committee of the Carlos III Health Institute (CEI PI 59_2020-v2).

## Results

A total of 2,067 individuals were included (Table 2). In both rounds, 50% of the participants were female. In R3 (November 2020), the mean age of participants was 46.1 years, 43.1% had a post-secondary level of education, and 56.7% were working. In R9 (November 2021), the participants’ mean age was 47.1 years, 31.8% of the participants had a post-secondary level of education, and 61.0% were working. Comparing R3 with R9, the proportion of participants who had COVID-19 increased by 32.2% (33.9% vs. 66.1%, respectively), and those who had family members or friends who had the disease increased by 13.0% (33.9% vs. 66.1%, respectively).

**TABLE 2:** Sociodemographic characteristics by round (Spain, 2020–2021).

		Round 3 *n* = 1,018		Round 9 *n* = 1,049		*p*-value^a^
		*n*	%	*n*	%	
Sex	Male	509	50.00	524	50.00	0.983
	Female	509	50.00	525	50.00	
	Total	1,018	100.00	1,049	100.00	
Age (years)	18–29	177	17.39	178	16.97	0.990
	30–44	301	29.57	307	29.27	
	45–60	336	33.01	353	33.65	
	>61	204	20.04	211	20.11	
	Total	1,018	100.00	1,049	100.00	
Education level	Primary	78	7.66	181	17.25	0.001
	Secondary	501	49.21	534	50.91	
	Post-secondary level	439	43.12	334	31.84	
	Total	1,018	100.00	1,049	100.00	
Employed	No	441	43,32	409	38.99	0.045
	Yes	577	56,68	640	61.01	
	Total	1,018	100.00	1,049	100.00	
Had COVID-19	No	949	93.22	915	87.23	0.001
	Yes	69	6.78	134	12.77	
	Total	1,018	100.00	1,049	100.00	
Relatives or friends with COVID-19	No	448	44.01	310	29.55	0.001
	Yes	570	55.99	739	70.45	
	Total	1,018	100.00	1,049	100.00	

^a^ Chi-square test.

**FIGURE 2:**
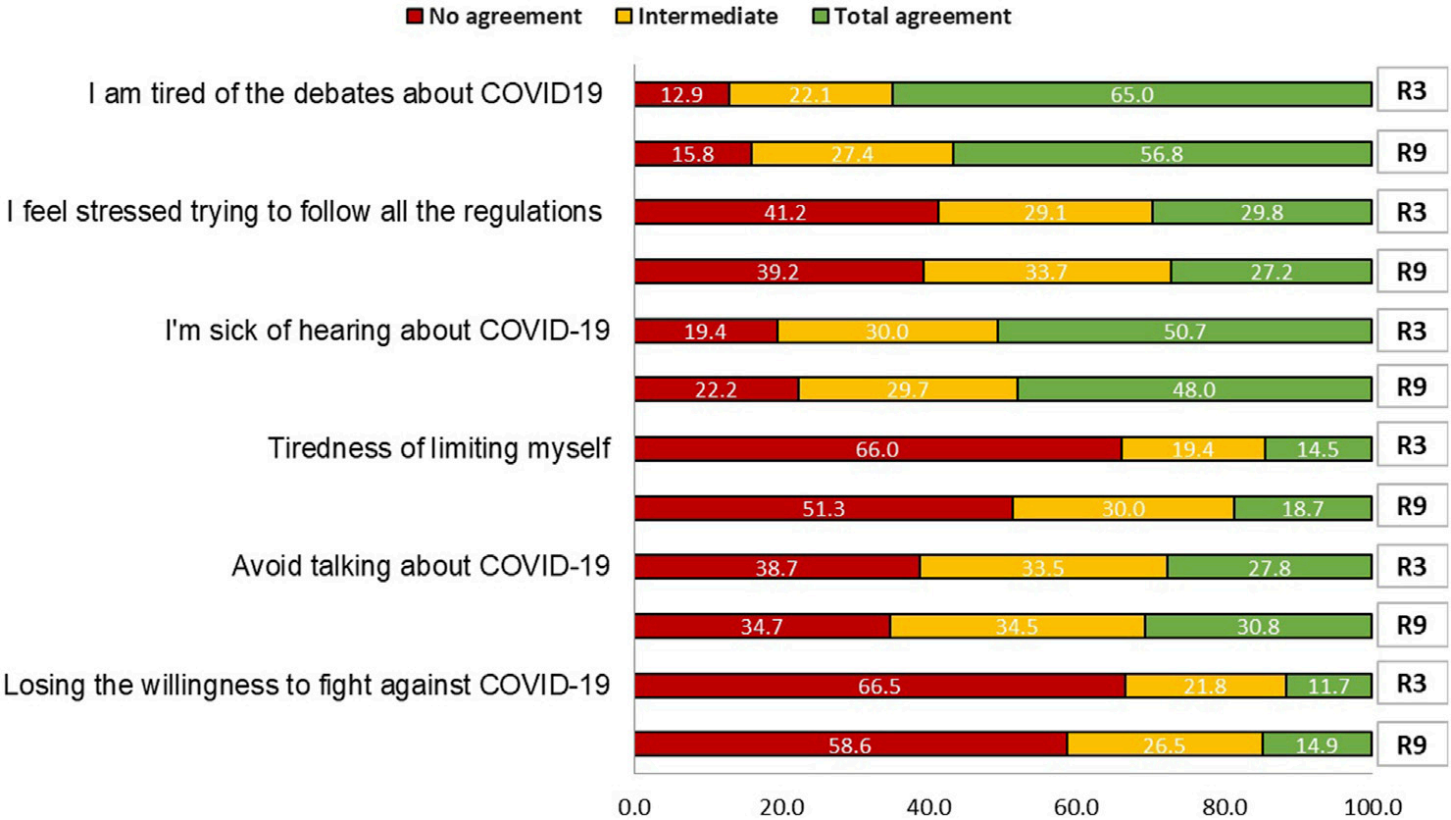
The COVID-19 Pandemic Fatigue Scale by rounds (Spain, 2020–2021). R3: Round 3, November 2020; R9: Round 9, November 2021.

Pandemic fatigue increased by 2.4% in 1 year (R3 mean 17.06 vs. R9 mean 17.47, *p* = 0.037). Regarding the scale components, being tired of limiting oneself to protect the most vulnerable and losing the will to fight COVID-19 increased across rounds by 4.20% (14.5% vs. 18.7%) and 3.20% (11.7% vs. 14.9%) respectively, showing a significant difference over time (*p* < 0.001). The response “I am tired of the debates about COVID-19” showed a decrease of 8.2% (65.0% vs. 56.8%) between rounds with a significant difference over time, and “I am sick of hearing about COVID-19” showed a significant decrease of 2.7% (50.7% vs. 48.0%) between rounds (Figure 2). The rest of the items showed no significant differences over time.

After 1 year, the knowledge of the population about COVID-19 prevention, transmission, and symptoms increased significantly (R3 mean 5.76 vs. R9 mean 6.39, *p* < 0.001) (Table 3). Attitudes towards COVID-19 changed during this time, and the feelings of depression and fear decreased significantly (means 3.21 vs. 2.95, *p* < 0.001, and means 3.20 vs. 3.10, *p* = 0.042, respectively).

**TABLE 3:** Knowledge, attitudes, concerns, perceptions, and practices by round (Spain, 2020–2021).

Variable (min-max)	Round 3		Round 9		
	Mean	Standard deviation	Mean	Standard deviation	*p*-value
Pandemic fatigue scale (6-30)	17.06	5.04	17.47	5.41	0.037
Knowledge about COVID-19					
Knowledge index (0–7)	5.76	0.91	6.39	1.20	<0.001
Attitudes towards COVID-19					
Feeling depressed (1–5)	3.21	1.20	2.95	1.22	<0.001
Feeling fearful (1–5)	3.20	1.21	3.10	1.22	0.042
Level of agreement with the decisions made scale (0–100)	60.00	17.00	49.00	23.00	<0.001
Consider that measures adopted are adequate (1–5)	2.64	1.28	2.98	1.20	<0.001
Consider that measures adopted are exaggerated (1–5)	2.20	1.16	2.24	1.15	0.309
Population concerns					
COVID-19 worry (1–5)	3.72	1.02	3.38	1.08	<0.001
Concerns scale (10–50)	37.74	7.29	36.37	8.21	<0.001
Risk perception					
Probability of becoming infected (1–5)	3.00	1.03	2.83	1.10	<0.001
Disease severity (1–5)	3.23	0.91	3.0	0.94	<0.001
Self-efficacy in avoiding becoming infected (1–5)	3.00	0.92	2.93	0.98	0.030
Feeling of rapid spread of the virus (1–5)	4.19	0.86	3.78	1.09	<0.001
Perception of the pandemic situation (1–5)	2.09	0.73	1.65	0.85	<0.001
Preventive practices					
Adherence to preventive measures scale (11–55)	46.29	6.57	41.69	8.42	<0.001
Intention to be vaccinated (1-5)	3.02	1.52	4.88	0.63	<0.001

Worries about COVID-19, the perceived probability of becoming infected, perceived severity of the disease, feeling that the coronavirus is rapidly transmitted, and self-efficacy decreased significantly over time (*p* < 0.001). Significant differences were also found in the perception of the pandemic situation (means 2.09 vs. 1.65, *p* < 0.001). Considering that the measures adopted by the government were adequate increased between rounds (*p* < 0.001) but the mean level of agreement with decisions taken by the authorities decreased significantly (means 60.0 vs. 49.0, *p* < 0.001). A 44% increase in intention to be vaccinated over time was found (means 3.02 vs. 4.88, *p* < 0.001) while adherence to preventive measures decreased significantly (Table 3).

### Change Between Rounds by Level of Pandemic Fatigue

**FIGURE 3:**
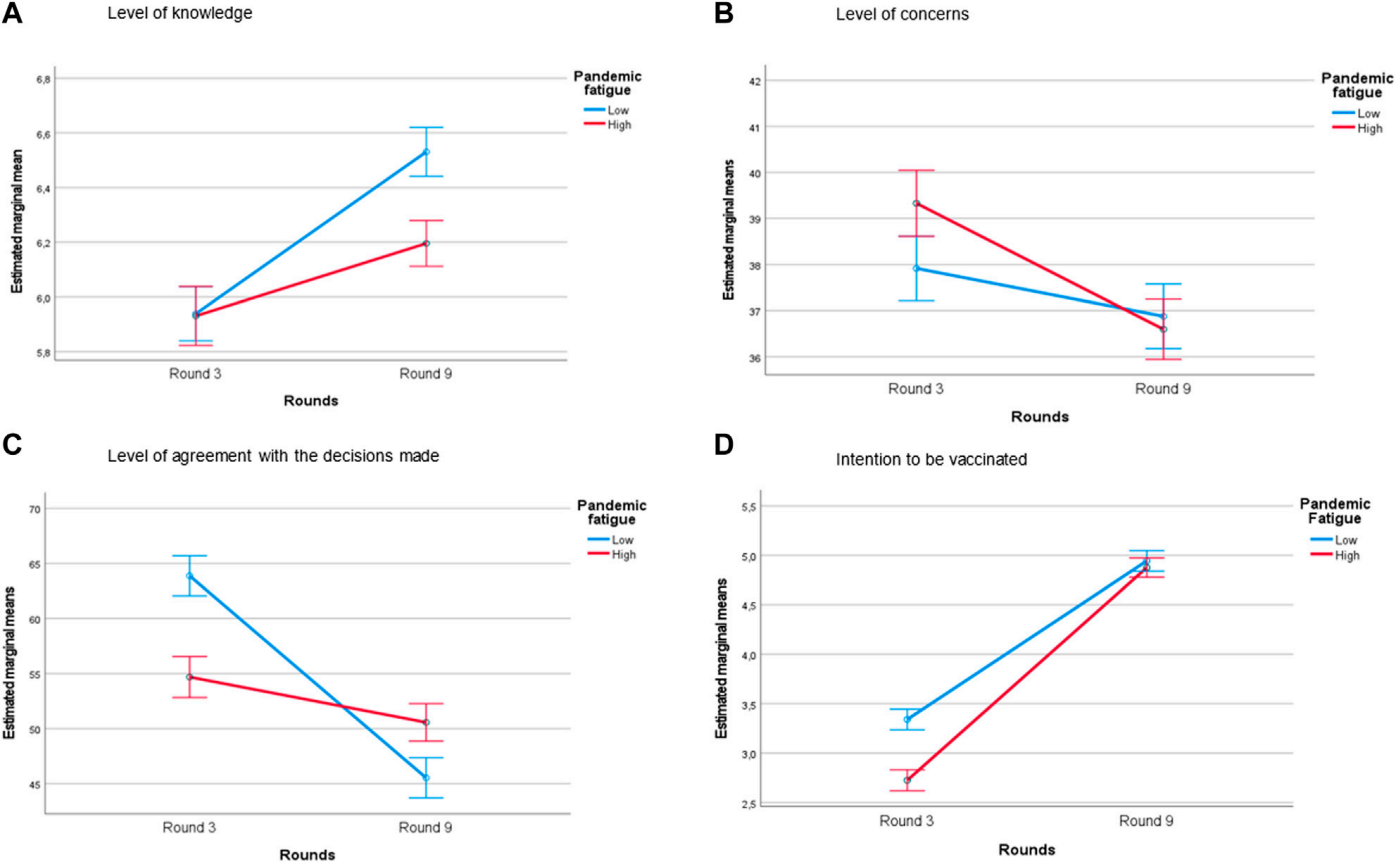
Knowledge, concerns, perceptions, and practices of individuals with different pandemic fatigue levels (high/low) during two rounds (margin plots of the significant interactions) (Spain, 2020–2021). Adjusted estimated marginal means from the general linear model with interaction between pandemic fatigue and rounds, adjusted for round, sex, job, age, and educational level. Only models with statistically significant interactions (*p* < 0.05) are shown in these margin plots.

The interactions between levels of pandemic fatigue and rounds were significant in the models for the following dependent variables: level of knowledge, concerns, agreement with the decisions made, and intention to be vaccinated, once adjusted by sex, age, education, and job (Figure 3).

There was an increase in the level of knowledge (Figure 3A) which was statistically significant between rounds for both groups (people with low and high levels of pandemic fatigue), although there was a greater increase in those with low levels of pandemic fatigue (estimated regression coefficient, *β* = 0.592; *p* < 0.001) compared with people with a high level of pandemic fatigue (*β* = 0.265; *p* < 0.001). No significant differences between low and high pandemic fatigue were found in R3 (*p* = 0.909) although there were differences in R9 (*p* < 0.001).

Figure 3B indicates the interaction of the model with the level of concern as the dependent variable. The decrease in the level of concern was statistically significant between rounds for both groups, being greater in those with high pandemic fatigue (*β* = −2.733; *p* < 0.001) than with low fatigue (*β* = −1.039; *p* = 0.029). In addition, initial levels of concern in R3 were significantly different between high and low levels of pandemic fatigue (*p* = 0.003), while in R9 the levels of concern were not significantly different between levels of pandemic fatigue (*p* = 0.553).

There was a significant decrease in the level of agreement with the decisions adopted between rounds for both the groups with high and low pandemic fatigue (Figure 3C). The interaction showed a higher decrease in the agreement of people with low pandemic fatigue (*β* = −18.346; *p* < 0.001) than in those with high fatigue (*β* = −4.121; *p* < 0.001). In R3, the level of agreement was significantly greater in those with low pandemic fatigue, while in R9, the level of agreement was higher in those with high pandemic fatigue (*p* < 0.001).

A significant increase between rounds in the intention to be vaccinated is observable in Figure 3D. The intention to be vaccinated had a greater increase between rounds in those with high pandemic fatigue (*β* = 2.150; *p* < 0.001) than those with low pandemic fatigue (*β* = 1.602; *p* < 0.001). Also, the initial intention to be vaccinated in R3 was higher in people with low fatigue than high fatigue (*p* < 0.001), although no significant difference was found 1 year later (R9) between those with low and high fatigue (*p* = 0.334).

## Discussion

This study shows significant differences in the knowledge, attitudes, concerns, perceptions, and vaccination intention during the first year of the COVID-19 pandemic in Spain and its interactions with pandemic fatigue level once adjusted for individual characteristics. The level of knowledge, concerns, agreement with the decisions made, and vaccination intention changed between rounds and levels of pandemic fatigue.

### Main Changes Between Rounds

Although cumulative incidence was high in both rounds, COVID-19 vaccines were not yet available in 2020, and hospital occupancy was twice as high as it was in 2021, when more than 89% of the population had received at least two doses of the COVID-19 vaccine [17]. Despite the relaxation of the most restrictive measures in November 2021, the main preventive measures to control the virus were still in place, such as maintaining social distancing, hand hygiene, and the use of facemasks.

Changes in knowledge, attitudes, concerns, perceptions, practices, and vaccination intention were observed between rounds. Knowledge about the COVID-19 increased significantly during the year of the study, while being depressed, fearful, or worried about COVID-19 decreased significantly following the improvement of the epidemiological situation. This evolution was explained by the belief that COVID-19 would soon be under control and by a growing confidence in overcoming the pandemic [29]. Likewise, COVID-19 risk perception (the probability of becoming infected and the disease severity) significantly decreased between rounds, as did adherence to preventive measures and the frequency of consulting information about the pandemic. However, the COVID-19 vaccination intention between these two rounds increased by 43% in 1 year [17]. At the beginning of the pandemic, COVID-19 vaccines generated much controversy due to their rapid development process, fears of health problems or lasting side effects, anti-vaccine beliefs, and the need for more information on the factors associated with not vaccinating [19, 20]. The high levels of acceptance of the COVID-19 vaccine among the population in Spain have had a great epidemiological impact on the decrease in hospitalisation and mortality rates [6, 21].

The level of agreement with the measures adopted in each round also decreased. In November 2020, the agreement with the restrictive measures adopted to prevent the expansion of COVID-19 was high. However, in November 2021, when the improvement of the epidemiological situation and the success of the vaccination campaign permitted a reduction in restrictions, meaning more social interaction, fewer preventive measures to follow, and greater mobility, the level of agreement of the population decreased. In general, the population supported regulations and, if anything, believed that they should have been stricter and introduced earlier [22]. 

### Pandemic Fatigue Evolution Between Rounds

Fatigue is a natural phenomenon that occurs over time due to a prolonged health crisis [23]. In November 2020, participants agreed with less than half of the items on the pandemic fatigue scale, while in November 2021, participants agreed with more than half of the items, reaching a mean of 17.47 points on a scale of 30. This implies that the Spanish population had, at that time, a moderate-to-high level of fatigue, in line with the level found in a previous study [5]. The items with the greatest increase in the level of fatigue after a year were “changing the subject when my friends or family talk about COVID-19”and “losing the will to fight against COVID-19.” As the COVID-19 pandemic affected our daily lives, our level of fatigue was expected to rise, the changes we made in our behaviour and perceptions produced an emotional cost that might be difficult to tolerate in the long term, so the willingness to fulfil these tasks should decrease [24]. Furthermore, the deficit between the amount of information in circulation and the cognitive capacity to assimilate such information together with the constant monothematic information led to oversaturation for the population [25]. This may influence the population to actively avoid information as the pandemic progresses [26]. Health authorities and media should provide accurate and concrete information to citizens to reduce avoidance behaviours, but not so much that it becomes overwhelming for the recipients that they lose interest [27].

### Interactions Between Pandemic Fatigue and Observed Changes in COVID-19 Knowledge, Attitudes, Concerns, and Vaccination Intention

Pandemic fatigue was not associated with knowledge about COVID-19 disease in November 2020, while 1 year later, despite the increase in knowledge observed during this period, we found an association between pandemic fatigue and knowledge: the more pandemic fatigue, the less the increase in disease knowledge, probably due to a reduction in the search for information. People with lower levels of knowledge and higher levels of fatigue are usually more vulnerable to the consequences of COVID-19 [28]. People tend to form accurate risk perceptions when the facts are known, and a lower level of knowledge can lead to a greater sense of threat, creating a cycle of distress [29]. Therefore, it is important to consider pandemic fatigue when it comes to providing clear and understandable information [30].

The level of concerns decreased in 1 year. In November 2020, those with high pandemic fatigue were more concerned than those with low fatigue, while 1 year later, this relation was reversed. It has being described how pandemic fatigue generates a phenomenon whereby individuals experience a decrease in COVID-19 concern over time despite if their real risk for infection remains stable or even increases [31]. At the beginning of the pandemic, the overcrowding of the healthcare system and the widespread fake news about the disease may have led to high uncertainty. Worrying about relatives or friends becoming infected with COVID-19 may lead to hypervigilance and distress [32]. When these concerns are sustained for a long time, feelings of extreme burden and exhaustion appear, resulting in fatigue, which leads to loss of strength and avoidance [7, 33]. Moreover, the avoidant response to the stressful situation leads to reducing negative thoughts, emotions, worries, and fears [34]. This would explain how the decrease in concerns was greater among those with high levels of pandemic fatigue because they might present a greater avoidance response as pandemic fatigue overrides individual differences in worries and caution [29]. Studying the effects of pandemic fatigue in the concerns will allow authorities to evaluate its negative effects in the risk perception of the population [31].

A higher level of agreement with the measures adopted in November 2020 in those with lower levels of pandemic fatigue has been found in other studies [28]. However, while the agreement with the measures decreased between rounds, it declined significantly more in those with lower fatigue levels than in those with higher fatigue. It seems that, despite the improvement of the epidemiological situation, people with lower levels of fatigue were more prone to adhere to strict preventive measures, while the higher-fatigued group probably welcomed the relaxation of measures implemented as it meant a reduction in their daily stress. Changes in preventive measures according to the pandemic situation have the potential to help control a pandemic, but it is only possible if they are fully accepted by the population [35].

It has been showed that pandemic fatigue could lead to higher rates of vaccination hesitancy [36]. In November 2020, before the vaccination campaign started, the intention to be vaccinated for COVID-19 was low in Spain, being significantly lower in those with higher pandemic fatigue. However, 1 year later, the intention to be vaccinated increased considerably but without significant association with the levels of fatigue in R9. This may be due to the willingness of both groups to see the end of the pandemic. The vaccination campaign in Spain managed to gain the trust of the population, contrary to what happened in other European countries where there is evidence of an increase in the refusal of vaccination in the same period [19]. The increase in the intention to be vaccinated may have been reinforced, among other factors, by the implementation of the vaccination card imposed in Spain in 2021 that gave access to social, cultural, and sportive events, as well as gymnasiums, hotels, and restaurants.

This study has some limitations. First, the cross-sectional nature of this data does not allow for the examination of causality, and the results may not be generalisable to other cultural contexts. Second, although the sample is representative of the Spanish population, responses to these surveys depended on having internet access. This may have left some vulnerable groups out of the study even though 96% of Spanish households had Internet access in 2021 [37]. In addition, it is possible that people with more pandemic fatigue were less willing to answer the questionnaire and thus the level of fatigue might be higher. However, the representative sample provides strength for the external validity of the study. Finally, most of the available scientific literature on pandemic fatigue was based on the first phase of the pandemic, which makes it difficult to compare our results from 2021.

### Conclusion

COVID-19 knowledge, attitudes, concerns, perceptions, practices, and vaccination intention changed after 1 year of the pandemic. Many of these changes could be affected by the context, the epidemiological situation, the control measures imposed, and showed a significant association with the levels of pandemic fatigue.

It is necessary to study the impact of pandemic fatigue beyond adherence to preventive behaviour and information search. That pandemic fatigue could also affect COVID-19-related knowledge and the level of concerns and agreement with the measures implemented is particularly relevant for public health strategies to consider these associations in the design of future interventions and awareness campaigns in the case of new waves and outbreaks.

Therefore, health authorities must choose the right moment to implement measures, taking into account population tiredness, and send messages, ensuring they are clear and concise to avoid overloading the population and reinforcing their resilience.
